# How the COVID-19 Pandemic Affected Attendance at a Tertiary Orthopedic Center Emergency Department: A Comparison between the First and Second Waves

**DOI:** 10.3390/diagnostics12112855

**Published:** 2022-11-18

**Authors:** Eleonora Carlicchi, Maria Eugenia Di Sabato, Antonino Cincotta, Riccardo Accetta, Alberto Aliprandi, Domenico Albano, Luca Maria Sconfienza, Carmelo Messina

**Affiliations:** 1 Department of Radiology, ASST Grande Ospedale Metropolitano Niguarda, 20162 Milan, Italy; 2 Department of Radiology, ASST Cremona, 26100 Cremona, Italy; 3 IRCCS Istituto Ortopedico Galeazzi, 20161 Milan, Italy; 4 Unità Operativa di Traumatologia, IRCCS Istituto Ortopedico Galeazzi, 20161 Milan, Italy; 5 Unit of Radiology, Clinical Institutes Zucchi, 20052 Monza, Italy; 6 Dipartimento di Scienze Biomediche per la Salute, Università degli Studi di Milano, 20122 Milan, Italy

**Keywords:** COVID-19, emergency department, orthopedic radiology

## Abstract

Italy was the first European country to face the SARS-CoV-2 virus (COVID-19) pandemic in 2020. The country quickly implemented strategies to contain contagions and re-organize medical resources. We evaluated the COVID-19 effects on the activity of a tertiary-level orthopedic emergency department (ED) during the first and second pandemic waves. We retrospectively collected and compared clinical radiological data of ED admissions during four periods: period A, first pandemic wave; period B, second pandemic wave; period C, three months before the COVID-19 outbreak; period D, same timeframe of the first wave but in 2019. During period A, we found a reduction in ED admissions (−68.2% and −59.9% compared with periods D and C) and a decrease in white codes (non-urgent) (−7.5%) compared with pre-pandemic periods, with a slight increase for all other codes: +6.3% green (urgent, not critical), +0.8% yellow (moderately critical) and +0.3% red (highly urgent, risk of death). We observed an increased rate of fracture diagnosis in period A: +14.9% and +13.3% compared with periods D and C. Our study shows that the COVID-19 pandemic caused a drastic change in the ED patient flow and clinical radiological activity, with a marked reduction in admissions and an increased rate of more severe triage codes and diagnosed fractures.

## 1. Introduction

The spread of the SARS-CoV-2 virus (COVID-19) pandemic around the world drastically impacted daily life activities, as well as medical practice [1,2,3]. 

Italy was the first European country to face the COVID-19 emergency and to experience the exponential growth of cases, which had a dramatic impact on the healthcare system and high mortality during spring 2020 (the “first wave”) [4]. Within Italy, Lombardy was undoubtedly the most affected region both at the beginning and throughout the pandemic, with about 38% of the Italian cases (and 48% of total deaths) [5,6]. After this period, in which Italy underwent a strict lockdown (8 March through 2 May 2020), the country continued to a summer with relatively low COVID-19 incidence and low mortality rate [7]. We then succumbed to a “second wave” from late August 2020, which reached a peak in late October 2020. During this second wave Italy re-adopted containment measures such as social distancing, personal protective equipment use and limitation of social activity. Nevertheless, the containment measures were somewhat less severe than the lockdown of the first wave [8].

The first wave of COVID-19 spread had catastrophic effects for the whole healthcare system, and several strategies have been implemented to meet the growing demand of intensive care unit beds and to contain the contagion [9]. The redistribution of health care resources aimed at increasing critical care capacity affected and disrupted clinical, surgical and research activity at many levels [10,11,12,13]. However, the need for ensuring care continuity did not stop emergency and trauma surgery, although they required a modulation of their activities to be adapted to the pandemic outbreak [14,15]. 

Similarly to other medical departments, radiology activity drastically changed during the COVID-19 outbreak, with a reported dramatic reduction in the amount of elective imaging examinations in the first wave [16,17,18]. In addition, radiology emergency practices acceded to a change in the type of exams that were predominantly performed; a situation that also reflected the change in population activities due to lockdown-forced social containment [19,20,21].

The primary outcome of our study is the evaluation of the COVID-19 outbreak impact on the activity of a tertiary-level orthopedic emergency department (ED) during the first and second waves, by integrating data from attendance flows with that of radiological activity and final diagnosis. Secondary outcomes were:

1. The comparison of first-wave data with the immediate period before the COVID-19 outbreak and the same timeframe of the first wave in 2019.
2. The comparison between the first and second waves of the pandemic.

## 2. Materials and Methods

### 2.1. Study Population

This retrospective study was performed at IRCCS Istituto Ortopedico Galeazzi, Milan, Italy. We collected and revised data from the ED electronic registry to obtain information about patient admission, combining it with that from the radiological department of our Institution. Emergency admissions were assessed during four periods:

- Period A: 21 February 2020–31 May 2020 (first wave of the pandemic)
- Period B: 1 October 2020–31 December 2020 (second wave of the pandemic)
- Period C: 1 December 2019–20 February 2020 (three months immediately before the COVID-19 outbreak)
- Period D: 21 February 2019–31 May 2019 (same timeframe of first wave of the pandemic spread but in 2019)

We retrospectively collected and classified the ED admissions during the four study periods by using the anonymized demographic, clinical and radiological data of admitted patients. The following variables were compared between periods:

- Age and sex
- Reason for admission (traumatic versus non-traumatic)
- Triage code at discharge: clinical severity according to four-degree urgency scale: white codes (non-urgent cases), green codes (urgent cases, not critical), yellow codes (moderately critical cases), red codes (highly urgent cases, risk of death). Admission triage code is assigned by paramedic staff according to a patient’s history and symptoms, whereas discharge triage code is assigned by the orthopedic surgeon at the end of the consultation (after the imaging study is performed, if needed).
- Discharge diagnosis (performed by the orthopedic surgeon).
- Discharge destination (home discharge, hospital admission, or voluntary discharge)

Inclusion criteria were mainly based on the ED access during the selected periods of the study, without age limits. We did not exclude pediatric patients from our analysis, in order to better assess the extent of ED admissions during the four periods. Regarding exclusion criteria, we did not include in our study patients with incomplete data or equivocal diagnosis. The analysis was limited to ED and radiological registries, without including laboratory test results.

### 2.2. Hospital Setting

Our hospital works as a tertiary-level orthopedic center with an ED specialized in minor traumatology. During the Italian lockdown (March–April 2020) our institution was classified as a hub for ‘minor’ trauma, accounting for single-district low-energy trauma that required orthopedic surgical treatment [10]. Additionally, the majority of outpatient and elective medical practice was suspended or postponed until 4 May, when Italy moved to “phase 2” of progressively reducing reopening of retail establishments, as well as allowing for increasing recreational activity [12].

In contrast to the first wave, during the second wave in our institution, we saw an attempt to keep elective activities open, also with a view to shortening waiting lists linked to the “loss” of previous months. This was achieved by ensuring control over admissions, including nasopharyngeal swabs that were performed before surgery.

### 2.3. Statistical Analysis

We performed a general descriptive analysis to compare the activity of the ED before and after the different periods of the COVID-19 outbreak. Data were reported as absolute values and percentages; continuous variables were expressed as mean and standard deviations (SD). The Shapiro–Wilk test was used to assess the normality of data distribution. Data from the first pandemic wave (period A) were compared to the other periods in terms of absolute number, absolute variations and relative variations. Absolute and relative variations were compared for statistical significance. Absolute variations refer to changes in absolute numbers between the two. Relative variations refer to changes when comparing the respective percentages of the periods. The chi-squared test of homogeneity or the Fisher’s exact test were used to assess the differences among groups for categorical variables (according to the sample size). The independent t-test was used for numerical variables. Statistical analysis was conducted using SPSS v24 (SPSS Inc., Chicago, IL, USA). A *p*-value < 0.05 was considered statistically significant.

## 3. Results

A total of 21,295 emergency traumatology admissions were analyzed: *n* = 2516 admissions were recorded during period A, *n* = 4595 during period B, *n* = 6278 during period C and *n* = 7906 during period D. Mean ± standard deviation of patients’ age according to the different periods was as follows: period A = 50.8 ± 23.2, period B = 50.2 ± 24, period C = 43.4 ± 23.1 and period D = 42.2 ± 23.4. No statistically significant differences were observed in terms of age between periods A and B (pandemic periods), as well as between periods C and D (non-pandemic periods); however, the mean age of subjects attending the ED department was significantly lower during periods C and D compared with periods A and B (*p* < 0.05).

A summary of access numbers, patient demographics, triage codes and discharge mode is reported in Table 1. The detailed comparison in terms of absolute and relative variations between the analyzed periods, according to data reported in Table 1, is reported in Table 2.

During the first pandemic wave (period A) we found a marked reduction of admissions to our ED: −68.2% (*p* < 0.05) compared with the same period in 2019 (period D) and −59.9% (*p* < 0.05) compared with the pre-pandemic period (period C). A significant reduction was also observed when comparing the first wave (period A) to the second wave (period B), where there was a reduction of 45.2% (*p* < 0.05) of admissions. Such remarkable changes in absolute variations of ED admissions inevitably affected the absolute changes in the remaining sub-analyses, which in most cases were statistically significant (see Table 2).

Regarding sex distribution, no statistically significant differences were observed between relative variation among different groups. Despite this, we found a slight prevalence of women attending the ED during the first and second waves (52.4% and 51.6%, respectively), whereas during the non-pandemic periods we found a slight male prevalence (51.8% in period C and 51.6% in period D).

**Figure 1:**
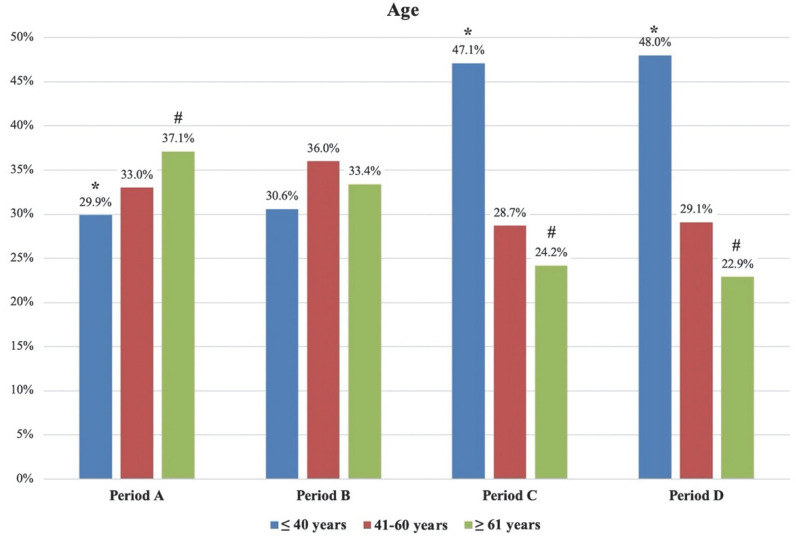
Visual comparison of the absolute percentages of patients’ age in the different periods. Period A: first pandemic wave; period B: second pandemic wave; period C: 1 December 2019–20 February 2020 (three months before the COVID-19 outbreak); period D: 21 February 2019–31 May 2019 (same timeframe of first wave but in 2019). * indicates statistical difference between period A and periods C/D for the group <40 years of age; # indicates statistical difference between period A and periods C/D for the group >61 years of age.

The mean age of patients was significantly higher during the first and second waves than in the non-pandemic periods (*p* < 0.05), whereas no statistically significant difference was observed between the first and second waves. A higher prevalence of patients over 61 years of age was observed during periods A and B (37.1% and 33.4%, respectively), compared with periods C and D (24.2% and 22.9%, respectively); the relative difference between periods A and C (+12.9%), as well as between periods A and D (+14.2%) was statistically significant. At the same time the prevalence of subjects younger than 40 years significantly decreased between the pandemic and non-pandemic periods (with a statistically significant relative variation of −17.2% between periods A and C and −18.2% between periods A and D). No significant changes were observed for the group of patients between 41 and 60 years of age. Figure 1 visually compares the absolute percentages of patients’ age in the different periods.

**Figure 2:**
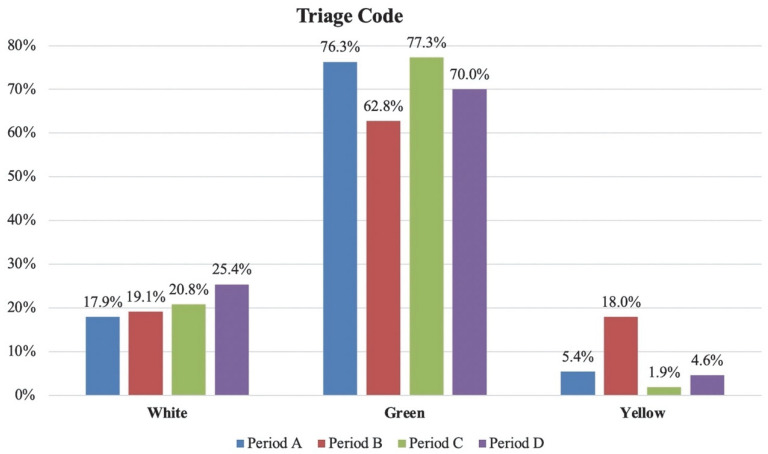
Visual comparison of the absolute percentages of triage code at discharge in the different periods. Period A: first pandemic wave; period B: second pandemic wave; period C: 1 December 2019–20 February 2020 (three months before the COVID-19 outbreak); period D: 21 February 2019–31 May 2019 (same timeframe of first wave but in 2019).

At discharge, a statistically significant reduction of −7.5% (relative variation) in white code was observed during the first wave (period A) compared with the same timeframe in 2019 (period D). A slight increase was observed for all other codes at discharge: +6.3% for green codes, +0.8% for yellow codes and +0.3% for red codes; none were statistically significant. When comparing the first and second waves, we found a considerable increase in yellow codes during the second wave (+12.6%, *p* < 0.05). This rate was significantly higher than all other periods. A statistically significant higher prevalence of green codes was observed during the first wave (+13.5%, *p* < 0.05). Figure 2 visually compares the absolute percentages of triage code at discharge in the different periods.

**Figure 3:**
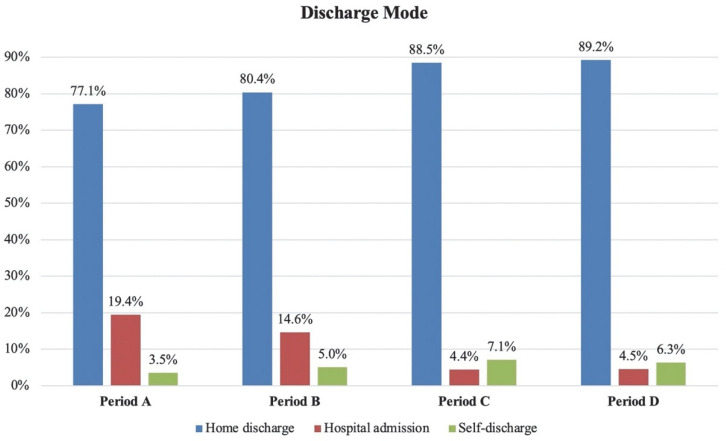
Visual comparison of the absolute percentages of t discharge destination in the different periods. Period A: first pandemic wave; period B: second pandemic wave; period C: 1 December 2019–20 February 2020 (three months before the COVID-19 outbreak); period D: 21 February 2019–31 May 2019 (same timeframe of first wave but in 2019).

We observed an increase in hospitalizations during the first and second waves, with a rate of hospital admissions of +19.4% during period A and 14.65% during period B, whereas it was +4.4% during period C and 4.5% during period D. The comparison of admissions between periods showed a relative variation of +4.8% (A vs. B, no significant difference), +15% (A vs. C, *p* < 0.05) and +14.9% (A vs. D, *p* < 0.05). The relative variation showed a statistically significant reduction in the rate of home discharge between periods A and C (−11.4%), as well as between periods A and D (−12.1%). Figure 3 visually compares the absolute percentages of discharge destination in the different periods.

A summary of the different diagnoses at discharge is reported in Table 3, which also considers the number of patients attending the ED for traumatic or non-traumatic reasons. The detailed comparison in terms of absolute and relative variations among diagnoses between periods is reported in Table 4.

A statistically significant increase in the number of patients discharged with a fracture was found when comparing period A and period D (+14.9%, *p* < 0.05), as well as between periods A and C (+13.3%, *p* < 0.05). Only a small increase was observed between the two waves (+1.8%), which was not significant.

Of note, an increase in the number of patients attending the ED for proximal femur fracture was observed during the pandemic waves. In fact, femoral fractures were *n* = 179/807 (19.7%) during period A, *n* = 262/1517 (16.7%) during period B, *n* = 77/1422 (5.4%) during period C and *n* = 99/1666 cases (5.9%) in period D. Taking the first wave as a reference, a statistically significant increase of +14.3% and +13.8% (periods C and D, respectively) was observed in the rate of proximal femoral fractures. No significant differences were observed between periods A and B.

**Figure 4:**
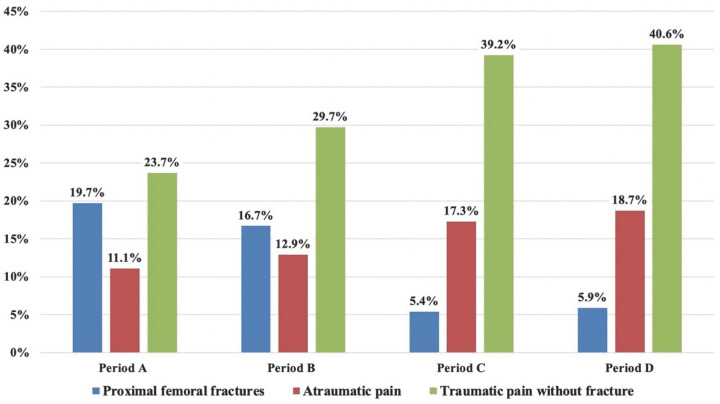
Visual comparison of the absolute percentages of proximal femoral fractures, atraumatic pain and traumatic pain without a fracture in the different periods. Period A: first pandemic wave; period B: second pandemic wave; period C: 1 December 2019–20 February 2020 (three months before the COVID-19 outbreak); period D: 21 February 2019–31 May 2019 (same timeframe of first wave but in 2019).

During the first wave we observed a marked reduction of those patients discharged with a diagnosis different from “fracture”. This was more evident for patients attending the ED with a history of trauma (such as contusions and sprains), with a statistically significant (*p* < 0.05) reduction during period A of −15.5% and −16.9% compared with periods C and D, respectively. This variation was mainly related to the significant decrease in the number of joint sprains. When considering non-fractured patients attending the ED for “atraumatic pain” (such as lower back pain, joint pain and malaise), we also observed an overall relative reduction during period A of −6.2% and −7.6% compared with periods C and D, respectively. Nevertheless, the relative difference was statistically significant only between periods A and D (*p* < 0.05). Figure 4 shows a visual comparison between the absolute percentages of proximal femoral fractures, atraumatic pain and traumatic pain without fracture, in the different periods.

The emergency-admissions data showed an overall increased rate of fracture diagnosis during the pandemic waves with a +36% (907) and +34.2% (1571) rate of X-rays showing fractures in period A and period B, respectively, compared with a rate of 22.7% (1422) and 21.1% (1666) of positive X-rays in period C and period D, respectively. The difference between pandemic periods (A and B) and non-pandemic periods (C and D) was statistically significant.

Considering each bone segment separately, a total number of *n* = 3963 X-rays were performed during the first wave, with a prevalence of positive findings of *n* = 2761 (69.7%). During the second wave, *n* = 6937 X-rays were performed, with a prevalence of positive findings of *n* = 3420 (49.3%). The percentage of positive X-rays was markedly lower during the non-pandemic time. During period C, a total number of *n* = 9538 X-rays were performed (*n* = 2680 with positive findings at 28.1%), whereas *n* = 12,022 X-rays were performed during period D (*n* = 3186 with positive findings at 26.5%). The difference between pandemic periods (A and B) and non-pandemic periods (C and D) was statistically significant.

**Figure 5:**
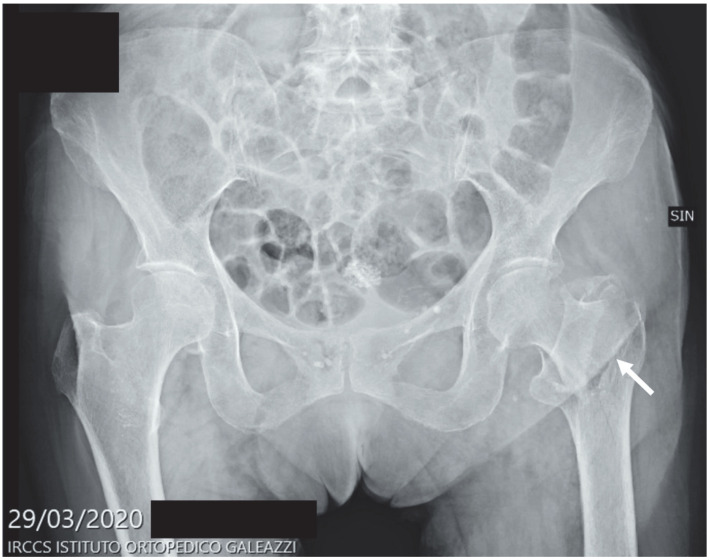
Figure shows a frontal pelvis X-ray examination of an 87-year-old female patient who suffered a left pertrochanteric femoral fracture (arrow) that has been surgically treated with an intramedullary nail.

**Figure 6:**
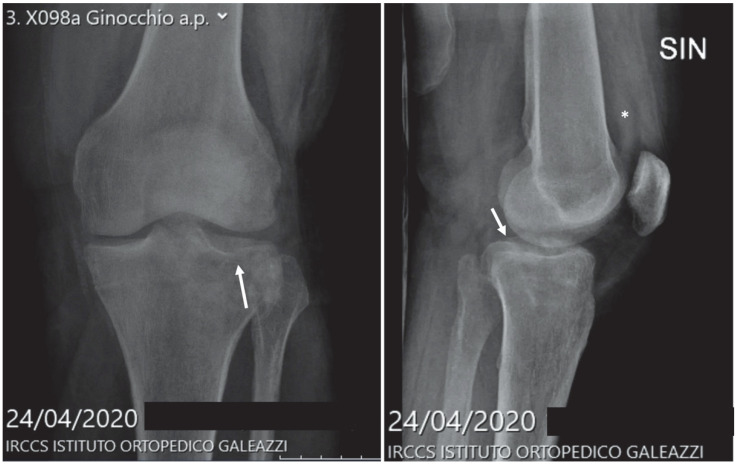
Figure shows a frontal and lateral knee X-ray examination of a 67-year-old male patient who suffered a non-displaced external left tibial plateau fracture (arrow) that has been treated conservatively with home-discharge after immobilization. Lateral X-ray shows mild knee post-traumatic effusion (asterisk).

**Figure 7:**
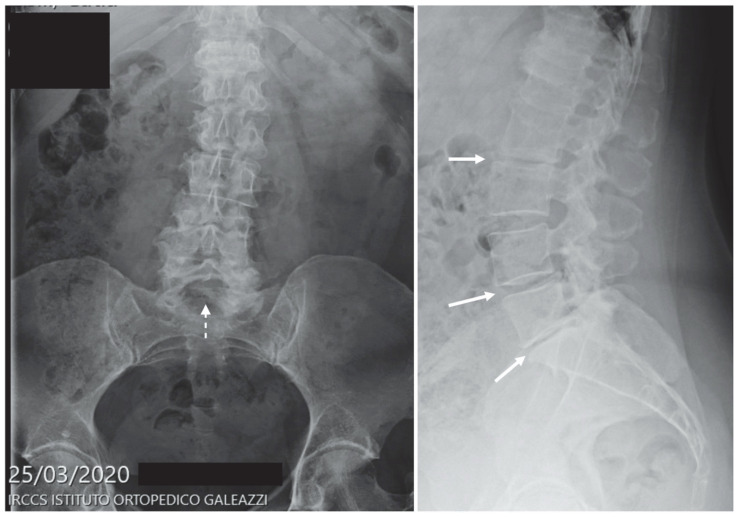
Figure shows a frontal and lateral lumbar spine X-ray examination of a patient suffering from sciatica, which presents with diffuse spondylarthrosis with lower tract spinal canal stenosis, mild scoliosis and loss of intervertebral disc space at multiple levels (arrows). Spina bifida occulta is also suspected at L5 (dashed arrow). The presence of vertebral fracture was excluded by follow-up MRI examination (not shown).

The following figures (Figure 5, Figure 6 and Figure 7) show three different radiographs of cases occurring during the first pandemic wave (period A): a proximal femur fracture (surgically treated after hospital admission), a tibial plateau fracture (conservatively treated with home discharge) and an X-ray of a patient suffering from sciatica (home discharge after pain relief prescription).

## 4. Discussion

The results of our study demonstrated that the COVID-19 pandemic caused a drastic change in the ED patient flow in the context of a tertiary-level orthopedic center. Similar results were also reported in a short report by Luceri et al. during the first pandemic wave [11] but were confirmed by also analyzing the pattern of admissions and diagnosis of the second COVID-19 pandemic wave.

First, we found a marked reduction of ED admissions. This was particularly evident during the first pandemic wave between March 2020 and May 2020, in which we found a decrease of about 68% compared with the same period of 2019 and a decrease of 60% compared with the pre-pandemic period. During the second wave we observed a lower—but consistent—reduction in ED admissions, of about 27% and 21% compared to 2019 and the pre-pandemic period, respectively. The main reason for such a difference is to be sought in the tight lockdown measures adopted during the first wave, which were eased progressively during the summer 2020 [7,22]. Lockdown limited population interaction and mobility, which surely reduced the risk of incurring trauma. At the same time, the fear of possible contagion made people with non-urgent conditions stay away from the ED [11,23].

Previous studies also reported a drastic reduction of ED admissions during the COVID-19 pandemic but were mainly focused on analyzing the first wave, which had the strongest impact on ED patient flow [10,11,24]. This may also be related to the increasing number of patients admitted into the intensive care units with severe disease, in which lower muscle mass contributed to a worse outcome for COVID-19 disease [25]. In our study, we also focused the analysis on the second pandemic wave, which was characterized by a series of non-pharmacological interventions (mask use, social distancing, etc.) similar to the first wave but less tight [7]. The consequence was a less pronounced reduction in ED admissions, configuring an intermediate scenario between the full lockdown and the non-pandemic periods.

Among the similarities between the first and second pandemic waves, we found a comparable distribution of patients’ mean age, which was significantly higher than in the non-pandemic periods. This was partly related to the highest concentration of fragility fractures in our ED during the COVID-19 spread, as well as for the likely lower incidence of trauma in the younger population due to reduced social mobility during the pandemic [11].

During the first pandemic wave we observed a reduction of the less urgent cases (the so-called walking wounded patients), with a decrease in the rate of white codes. At the same time, admission triage rates of yellow and red codes noticeably increased during the two pandemic waves. A reduction in such scenarios has been documented not only during the COVID-19 pandemic [10,11] but also during the SARS epidemic in 2003 [26]. As stated above, this may also be a consequence of people’s perception of an ED as a place at risk of infection. On the other hand, there are also papers describing mixed situations. For example, Hahn et al. report no significant difference in the admissions for minor trauma during 2020 compared with the years prior to the COVID-19 pandemic (2017–2019) [27]. For greater clarity, the discharge diagnosis of “malaise” includes patients presenting with general symptoms of physical discomfort and dizziness (with or without syncope) but without imaging evidence of critical conditions (e.g., without evidence of bleeding or stroke at CT scan). As a matter of fact, our hospital a third-level orthopedic center, more urgent neurological or cardiovascular cases are usually diverted to the ED department of neighboring hospitals.

Despite many similarities, during the second wave we observed a considerable increase in the rate of yellow codes, which was significantly higher than all other periods including the first wave.

A clear example is represented by the significant increase in the absolute and relative rate of proximal femur fracture observed during the pandemic waves. This can be explained by the fact that our center was designated by regional regulations as a hub for middle to intermediate severity trauma, including fragility fractures. Compared with vertebral fractures that may be under diagnosed, proximal femur fractures typically require immediate hospitalization, providing a unique snapshot of the situation of fragility fractures in the pandemic months [10]. Our results differ from those of the study by Runtz et al., in which a decrease in the number of pelvic fragility fractures was observed in older patients during the lockdown [28]. Similarly, Wong et al. found a significant a decrease in hip fractures of about 20% during the COVID lockdown periods compared with the same periods in 2016. The increase in femoral fracture we found can be explained by the specific organization of our hospital, in which patients suffering from fragility fractures were specifically addressed.

This hub setting in the first pandemic wave was further strengthened in the second wave and the epidemiological change in the type of patients and fractures was confirmed, with admission of patients with higher mean age and suffering mainly from femoral fractures.

Regarding radiology, during the pandemic waves we observed an increase in the percentage of fractures detected at radiographic examinations in the ED. Such an increase, as for other settings, may be the consequence of a possible fear of contagion by patients, resulting in fewer non-fractured subjects attending the ED [11]. This also had implications in the overall prevalence of positive findings at X-rays, with the percentage of positive X-rays markedly higher during the pandemic waves.

Our study carries the limitations of a retrospective study, as well as the fact that we present data only from a single center which were extracted a posteriori. It is therefore possible that certain aspects of the comparison between the two pandemic waves have not been captured, especially when considering other, not purely orthopedic, settings. Nevertheless, we emphasize the fact that our institution represents one of the biggest orthopedic hospitals in Northern Italy, therefore still provides informative data from a wide local area. Another limitation is that we did not include in our analysis data from laboratory tests, which in some cases could have provided useful information for more precise diagnoses.

## 5. Conclusions

In this work we report, for the first time, the experience of a third-level orthopedic ED in Northern Italy during the first two waves of the SARS-CoV-2 pandemic, comparing these periods with previous non-pandemic periods. Data from the first two waves of the COVID-19 pandemic were unique due to the lack of vaccination coverage in the population, therefore offer a specific view on the very early pandemic months. The first two waves showed similarities in terms of those fractures addressed to the hub system of our hospital, where organization improved over time. On the other side, the easing of strict lockdown measures in the time between the first and second waves, which were partly maintained during the second wave, led to some similarities re-emerging between the second wave and non-pandemic periods [29]. Despite similar trends of sudden reduced activity having been already extensively reported in other clinical scenarios, we emphasize that to the best of our knowledge no study has focused on a setting of a third-level orthopedic center. Data from our study may be of further use in the future to be considered in view of specific needs during similar scenarios.

## Figures and Tables

**Table 1:** Summary of access numbers according to patient demographics, triage codes and discharge mode according to the different periods. ED = Emergency Department.

	Period A (1st Wave) 21 February 2020–31 May 2020		Period B (2nd Wave) 1 October 2020–31 December 2020		Period C 1 December 2019–20 February 2020		Period D 21 February 2019–31 May 2019	
	*n*=	%	*n*=	%	*n*=	%	*n*=	%
**Total ED Admissions**	2516		4595		6278		7906	
**Gender**								
Males	1197	47.6%	2222	48.4%	3251	51.8%	4080	51.6%
Females	1319	52.4%	2373	51.6%	3027	48.2%	3826	48.4%
Age								
≤40 years	751	29.9%	1406	30.6%	2958	47.1%	3800	48.0%
41–60 years	831	33.0%	1655	36.0%	1801	28.7%	2299	29.1%
≥61 years	934	37.1%	1534	33.4%	1519	24.2%	1807	22.9%
**Triage Code**								
White	450	17.9%	877	19.1%	1304	20.8%	2007	25.4%
Green	1921	76.3%	2884	62.8%	4855	77.3%	5536	70.0%
Yellow	136	5.4%	827	18.0%	118	1.9%	362	4.6%
Red	9	0.4%	7	0.2%	1	0.02%	1	0.01%
**Discharge**								
Home discharge	1939	77.1%	3694	80.4%	5558	88.5%	7053	89.2%
Hospital admission	489	19.4%	673	14.6%	276	4.4%	357	4.5%
Self-discharge	88	3.5%	228	5.0%	444	7.1%	496	6.3%

**Table 2:** Detailed comparison between period A and periods B/C/D in terms of absolute and relative variations in terms of patient demographics, triage codes and discharge mode. Absolute variations refer to changes in absolute numbers between the two periods (e.g., total number A vs. total number B). Relative variations refer to changes when comparing the respective percentages of periods (e.g., percentage A vs. percentage B). ED = Emergency Department. An asterisk (*) highlights statistically significant differences (*p* < 0.05) between the groups.

	Comparison between Periods A and B		Comparison between Periods A and C		Comparison between Periods A and D	
	Absolute Variation	Relative Variation	Absolute Variation	Relative Variation	Absolute Variation	Relative Variation
Total ED Admissions	−45.2% (*)		−59.9% (*)		−68.2% (*)	
**Gender**						
Males	−46.1% (*)	−0.8%	−63.2% (*)	−4.2%	−70.7% (*)	−4.0%
Females	−44.4% (*)	+0.8%	−56.4% (*)	+4.2%	−65.5% (*)	+4.0%
**Age**						
≤40 years	−46.6% (*)	−0.7%	−74.6% (*)	−17.2% (*)	−80.2% (*)	−18.2% (*)
41–60 years	−49.8% (*)	−3.0%	−53.9% (*)	+4.3%	−63.9% (*)	+3.9%
≥61 years	−39.1% (*)	+3.7%	−38.5% (*)	+12.9% (*)	−48.3% (*)	+14.2% (*)
**Triage Code**						
White	−48.7% (*)	−1.2%	−65.5% (*)	−2.9%	−77.6% (*)	−7.5% (*)
Green	−33.4% (*)	+13.5% (*)	−60.4% (*)	−1.0%	−65.3% (*)	+6.3%
Yellow	−83.6% (*)	−12.6% (*)	−15.3% (*)	+3.5%	−62.4% (*)	+0.8%
Red	+28.6% (*)	+0.2%	+800.0% (*)	+0.3%	+800.0% (*)	+0.3%
**Discharge**						
Home discharge	−47.5% (*)	−3.3%	−65.1% (*)	−11.4% (*)	−72.5% (*)	−12.1% (*)
Hospital admission	−27.3% (*)	+4.8%	+77.2% (*)	+15.0% (*)	+37.0% (*)	+14.9% (*)
Self-discharge	−61.4% (*)	−1.5%	−80.2% (*)	−3.6%	−82.3% (*)	−2.8%

**Table 3:** Summary of access numbers according to the different diagnoses at discharge. ED = Emergency Department.

	Period A (1st Wave) 21 February 2020–31 May 2020		Period B (2nd Wave) 1 October 2020–31 December 2020		Period C 1 December 2019–20 February 2020		Period D 21 February 2019–31 May 2019	
**Total ED Admissions**	2516		4595		6278		7906	
Total fractures	907	36.0%	1571	34.2%	1422	22.7%	1666	21.1%
Proximal femoral fractures	179	19.7%	262	16.7%	77	5.4%	99	5.9%
**Atraumatic pain**								
Lower back pain	70	2.8%	171	3.7%	303	4.8%	384	4.9%
Sciatica	36	1.4%	96	2.1%	142	2.3%	242	3.1%
Joint pain	109	4.3%	226	4.9%	421	6.7%	600	7.6%
Malaise	33	1.3%	38	0.8%	104	1.7%	127	1.6%
Osteoarthritis	22	0.9%	40	0.9%	80	1.3%	91	1.2%
Tendinitis	10	0.4%	22	0.5%	35	0.6%	31	0.4%
Total	280	11.1%	593	12.9%	1085	17.3%	1456	18.7%
**Traumatic pain without fracture**								
Bone contusion	253	10.1%	655	14.3%	898	14.3%	1087	13.7%
Joint sprain	343	13.6%	712	15.5%	1561	24.9%	2123	26.9%
Total	596	23.7%	1367	29.7%	2459	39.2%	3210	40.6%

**Table 4:** Detailed comparison between period A and periods B/C/D in terms of absolute and relative variations in terms of the different diagnoses at discharge. Absolute variations refer to changes in absolute numbers between the two periods (e.g., total number A vs. total number B). Relative variations refer to changes when comparing the respective percentages of periods (e.g., percentage A vs. percentage B). ED = Emergency Department. An asterisk (*) highlights statistically significant differences (*p* < 0.05) between the groups.

	Comparison between Periods A and B		Comparison between Periods A and C		Comparison between Periods A and D	
	Absolute Variation	Relative Variation	Absolute Variation	Relative Variation	Absolute Variation	Relative Variation
**Total ED Admissions**	−45.2% (*)		−59.9% (*)		−68.2% (*)	
Total fractures	−42.3% (*)	+1.8%	−36.2% (*)	+13.3% (*)	−45.6% (*)	+14.9% (*)
Proximal femoral fractures	−31.7% (*)	+3.0%	+132.5% (*)	+14.3% (*)	+80.8% (*)	+13.8% (*)
**Atraumatic pain**						
Lower back pain	−59.1% (*)	−0.9%	−76.9% (*)	−2.0%	−81.8% (*)	−2.1%
Sciatica	−62.5% (*)	−0.7%	−74.6% (*)	−0.9%	−85.1% (*)	−1.7%
Joint pain	−51.8% (*)	−0.6%	−74.1% (*)	−2.4%	−81.8% (*)	−3.3%
Malaise	−13.2% (*)	+0.5%	−68.3% (*)	−0.4%	−74.0% (*)	−0.3%
Osteoarthritis	−45.0% (*)	0%	−72.5% (*)	−0.4%	−75.8% (*)	−0.3%
Tendinitis	−54.5% (*)	−0.1%	−71.4% (*)	−0.2%	−67.7% (*)	0%
Total	−52.8% (*)	−1.8%	−74.2% (*)	−6.2%	−80.8% (*)	−7.6% (*)
**Traumatic pain without fracture**						
Bone contusion	−61.4%	−4.2%	−71.8%	−4.2%	−76.7%	−3.6%
Joint sprain	−51.8%	−1.9%	−78.0%	−11.3% (*)	−83.8%	−13.3% (*)
Total	−56.4%	−6.0%	−75.8%	−15.5% (*)	−81.4%	−16.9% (*)

## Data Availability

The data presented in this study are available on request from the corresponding author.
